# Prostate stromal sarcoma mimicking benign prostate hyperplasia: A case report

**DOI:** 10.1016/j.ijscr.2024.110088

**Published:** 2024-07-27

**Authors:** Fauriski Febrian Prapiska, Rizky An Nabil, Syah Mirsya Warli, Bungaran Sihombing, Dhirajaya Dharma Kadar, Ginanda Putra Siregar

**Affiliations:** a Division of Urology, Department of Surgery, Faculty of Medicine, Universitas Sumatera Utara – Haji Adam Malik General Hospital, Medan, Indonesia; b Department of Urology, Faculty of Medicine, Universitas Sumatera Utara – Universitas Sumatera Utara Hospital, Medan, Indonesia; c Department of Urology, Faculty of Medicine, Universitas Indonesia – Haji Adam Malik General Hospital, Medan, Indonesia

**Keywords:** Urology, Prostate, Benign prostate hyperplasia

## Abstract

**Introduction & objectives:**

Prostate stromal sarcoma is extremely rare and aggressive malignancy accounting for less than 1 % of all type of prostate cancers. It is frequently misdiagnosed from other lower urinary tract symptoms (LUTS) problems.

**Case presentation:**

We present a case report of 45-year-old male complaining with LUTS problems. Patient also suffers anorexia and weight loss. He was first diagnosed with benign prostate hyperplasia (BPH). Patients had done transurethral resection of prostate (TURP) to alleviate the complaint, but the symptoms worsened and recurred. Histopathological examination findings confirmed prostate stromal sarcoma (T4N0M0). Patient was further examined using MRI and then radical prostatectomy procedure was performed.

**Discussion:**

Incidence of prostate stromal sarcoma is very low and most commonly presents with obstructive LUTS symptoms. This could mimic other disease such as BPH or other type of prostate cancer. Therefore, clinicians require a high suspicion in patient with recurrent LUTS.

**Conclusion:**

Prostate stromal sarcoma diagnosis is a challenging disease entity that necessitates histopathology examination. Timely and accurate diagnosis of prostate stromal sarcoma is needed to achieve better outcome and prognosis for the patients.

## Highlights

• Prostate stromal sarcoma is an extremely rare and aggressive malignancy, constituting less than 1 % of all prostate cancers. It is often misdiagnosed due to its similarity to other lower urinary tract symptoms (LUTS) problems.
• Here we present a 45-year-old male presented with LUTS complaints, accompanied by anorexia and weight loss. Initially diagnosed with benign prostate hyperplasia (BPH) and underwent transurethral resection of the prostate (TURP), which failed to resolve symptoms.
• Histopathological examination confirmed prostate stromal sarcoma (T4N0M0). Further evaluation with MRI revealed invasion into the urinary bladder, prompting radical prostatectomy.
• Prostate stromal sarcoma commonly presents with obstructive LUTS symptoms, mimicking other conditions like BPH or prostate cancer.
• Prostate stromal sarcoma poses diagnostic challenges, requiring histopathological examination for accurate diagnosis. Timely and accurtate diganosis is crucial for better patient outcome and prognosis

## 1 Introduction

The range of potential prostate abnormalities is extensive and can be divided into common and less common conditions. The common conditions include acinar adenocarcinoma, benign prostatic hyperplasia, chronic prostatitis, hemorrhage, cysts, calcifications, atrophy, and fibrosis. On the other hand, the less common conditions encompass tumors other than acinar adenocarcinoma, granulomatous prostatitis involving tuberculosis, abscesses, idiopathic disorders like amyloidosis, and exophytic benign prostatic hyperplasia [1]. Prostatic sarcoma is a rare form of cancer since the prostate mainly consists of glandular tissue, with adenocarcinomas being the most malignant tumors [2]. Only around 5 % of all soft tissue sarcomas affect the genitourinary tract [3]. One type of soft tissue sarcoma is stromal sarcoma, which primarily affects young adults and is often found in the para-articular region of the extremities. Prostate stromal sarcoma is an exceedingly uncommon and aggressive malignancy, making up less than 0.1 % of occurrences [4,5]. Due to its vague presentation and rarity, stromal sarcoma of the prostate is usually diagnosed at an advanced stage and is frequently mistaken for other lower urinary tract symptoms (LUTS) issues.

## 2 Methods

This work has been meticulously conducted and reported in accordance with the SCARE (Surgical Case Report) guidelines, ensuring that all aspects of the case and the methodology employed are transparently and comprehensively documented [21]. We have adhered to the criteria set forth by SCARE to facilitate the accurate, complete, and transparent reporting of this surgical case study.

## 3 Case presentation

**Fig. 1:**
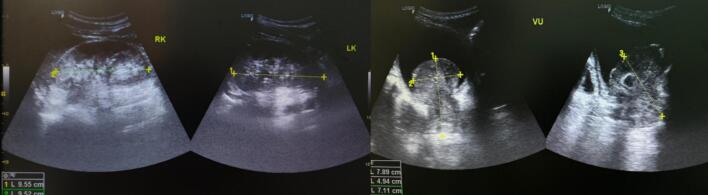
Ultrasound examination.

**Fig. 2:**
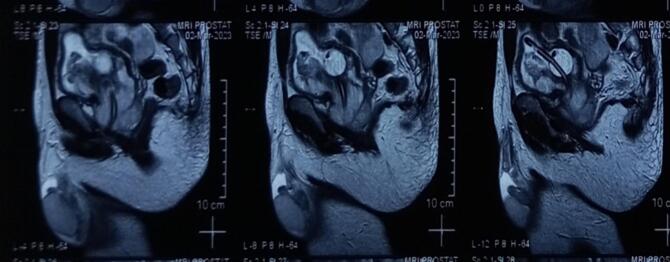
MRI examination.

A 45-year-old male patient was admitted to the hospital with chief complaint of urinary retention. The patient has experienced a weight loss of 7 kg over the past two months, and his appetite has decreased. He was first diagnosed with benign prostate hyperplasia (BPH). Patients had done transurethral resection of prostate (TURP) to alleviate the complaint, but the symptoms worsened and recurred. Due to the persistence of symptoms, the patient underwent further investigations including ultrasound (Fig. 1) and MRI (Fig. 2). He was diagnosed with prostate tumor1. The tumor had been treated with laser ablation three times before.

**Fig. 3:**
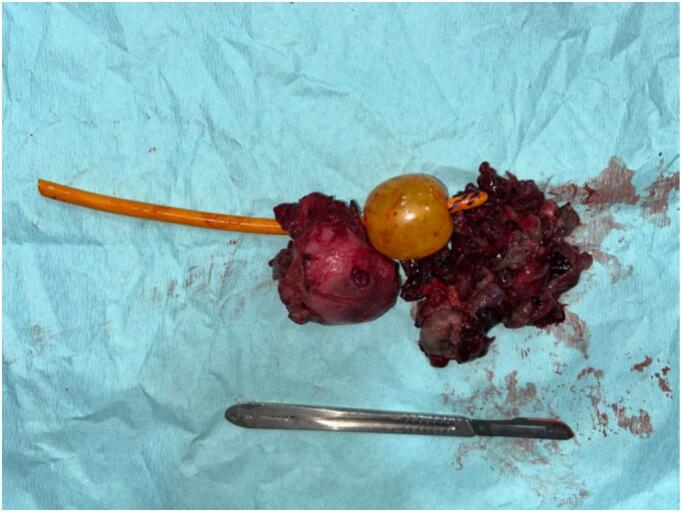
Gross specimen.

**Fig. 4:**
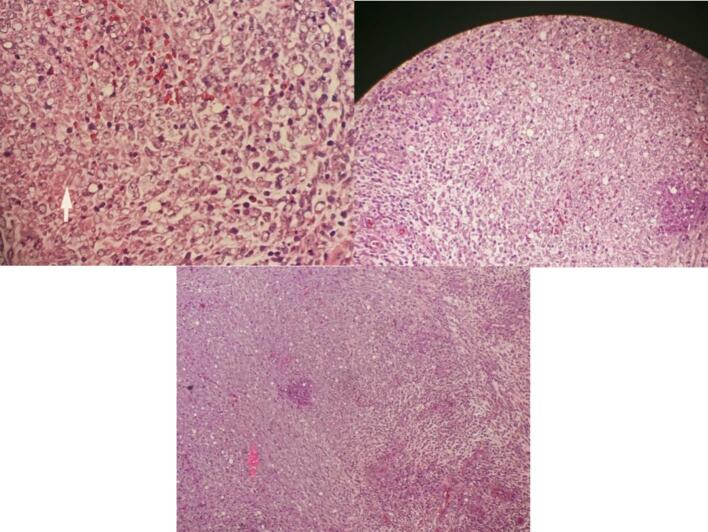
H&E staining shows prostate sarcoma image (100× magnification).

The MRI results indicate that there is a malignant mass in the prostate that extends into and infiltrates the urinary bladder. This invasion leads to a narrowing of the urethra. However, there are no signs of metastases based on the staging, which is classified as T4N0M0. The patient underwent radical prostatectomy, and the specimen was taken to be examined (Fig. 3). From the results of anatomic pathology, it appears that prostate tissue consists of proliferating atypical and hypercellular mesenchymal cells. Some infiltrate between benign prostate glands to form storiform, fibrosacomatous, leaf-like, phylodes-like structures. Cells with a spindle-shaped nucleus, hyperchromatism, eosinophilic cytoplasm (Fig. 4). Glands of varying shape and size with 2 layers of epithelium, the lumen is lined with columnar epithelium which undulates into the lumen (papillary infoldings) and cuboidal cells in the basal layer. Mitosis and necrosis are found. Based on the anatomic pathology findings, the diagnosis is prostatic stromal sarcoma.

## 4 Discussion

Prostatic stromal sarcoma is a rare type of cancer characterized by abnormal proliferation of stromal cells within the prostate gland [5]. Typically affecting older adults, its symptoms can mirror benign prostate conditions, making diagnosis challenging. The main presenting symptom in our patient was urinary retention, likely caused by the tumor's growth compressing the urethra and invading the urinary bladder. Unintentional weight loss and decreased appetite may be systemic manifestations of the malignancy. As of the current knowledge available in the academic medical literature, the documented incidence of intraprostatic stromal sarcoma remains limited, comprising a total of 15 reported cases (including this case report) [2,4,7,8,[10], [11], [12], [13], [14], [15], [16], [17], [18]]. Analysis of this reported cohort reveals a mean age of 41.4 years at the time of diagnosis, with patients ranging from 22 to 63 years old. It is worth noting that prostatic sarcoma tends to manifest predominantly in a younger demographic, distinct from the prevailing malignancy of the prostate, adenocarcinoma [19].

A majority of patients afflicted with prostatic sarcoma typically present with indications of bladder outlet obstruction and commonly exhibit various nonspecific lower urinary tract symptoms, such as dysuria, heightened urinary frequency, and nocturia. Less frequently encountered presenting symptoms encompass hematuria, micturition, constipation, and discomfort in the pelvic, perineal, rectal, and back regions. It has been documented that the elevation of prostate-specific antigen (PSA) levels may not occur in patients with prostatic sarcoma due to the tumor's non-epithelial origin [19]. Previous cases of prostatic stromal sarcoma have demonstrated PSA levels ranging from 0.35 ng/ml to 5.94 ng/ml. In this patient's PSA level was 2.7 ng/ml.1 Tumor size usually varies from 5.5 to 14 cm, generally exceeding the dimensions of prostate epithelial lesions. The imaging characteristics of primary stromal sarcoma of the prostate lack distinctiveness. For instance, a case report by Shirakawa [16] revealed a high signal mass originating from the prostatic fascia in T2-weighted MRI. Nonetheless, identifying the tumor's origin solely based on MRI findings remains challenging, necessitating histopathological examination for a definitive diagnosis. Typical findings associated with stromal sarcoma involve positive expression of vimentin, cytokeratin, CK7, CD99, Bcl-2, SS18-SSX2, and SYT-SSX in the tumor cells. Notably, the presence of SS18-SSX gene fusion serves as a crucial marker for diagnosing prostate stromal sarcoma [20].

Due to the tumor's highly malignant nature, patients afflicted with this condition experience a remarkably brief survival period, and the disease generally demonstrates a rapid progression. The tumor spreads via two primary mechanisms: systemic dissemination and local invasion. Previous cases have displayed varying degrees of invasion into nearby structures, such as the seminal vesicle glands, cavernous body of the penis, bladder, and rectum. This invasive behavior can lead to symptoms such as dysuria. Additionally, metastasis to regional lymph nodes, liver, and lungs has been observed. Despite the implementation of multimodal treatment approaches, the average survival time for affected individuals is approximately 24 months [8].

The management of this sarcoma remains uncertain primarily due to the limited experience with this rare condition. However, it seems that aggressive surgical resection is a crucial consideration for treating locally confined stromal sarcoma [17]. The potential benefits and efficacy of adjuvant chemotherapy and/or radiation therapy have not been clearly defined, but their utilization is strongly advised given the unfavorable prognosis associated with this malignancy. To advance our understanding and establish more appropriate therapeutic approaches, further documentation and analysis of additional cases are warranted.

When encountering a relatively young male patient with a sizeable and well-defined tumor in the prostate, and the PSA level is within the normal range, stromal sarcoma becomes a potential consideration in the differential diagnosis, particularly when imaging features reveal increased signal intensity on T2-weighted MRI images and mild heterogeneous contrast enhancement [4]. In the clinical diagnosis of prostate stromal sarcoma, it is essential to first rule out the possibility of metastatic stromal sarcoma. In this case, the initial symptoms were related to urinary system disturbances. A thorough clinical examination, including MRI, did not detect any other tumors in the body apart from the prostate lesion. Additionally, the likelihood of sarcomatoid carcinoma should be taken into account when dealing with sarcomatoid tumors. Immunohistochemical analysis of epithelial markers may exhibit diffuse positivity. Furthermore, compared to prostate cancer, prostate stromal sarcoma typically occurs at a younger age and is associated with normal serum PSA levels. Stromal sarcoma tends to manifest with rapid onset and progression, often presenting as a large tumor with distinct boundaries in relation to the prostate. Moreover, conducting a physical examination in the anus can be highly beneficial in distinguishing prostate stromal sarcoma from prostate cancer. In the case of prostate sarcoma, the mass is usually sizable and causes discomfort to the patient due to tumor enlargement [10].

## 5 Conclusion

Prostatic stromal sarcoma, particularly in the form of stromal sarcoma, is a rare and aggressive malignancy that primarily affects younger individuals, usually presenting with urinary symptoms and a normal PSA level. Due to its limited incidence and distinct clinical characteristics, accurate diagnosis and appropriate management remain challenging. Further research and documentation of cases are necessary to establish effective therapeutic strategies for this uncommon type of cancer.

## Patient perspective

“I didn't expect to have a prostate tumor. It was truly shocking when I first heard about the diagnosis, although my partners had mentioned that my declining weight could be a sign of cancer. At first, it was hard to have a complete feeling after urinating, and eventually, I couldn't urinate anymore. The doctor said that my tumor had not metastasized yet, and I hope it can be completely treated. I'm willing to undergo any surgeries needed to remove the cancer from my body.”

## Consent

Written informed consent was obtained from the patient for publication of this case report and accompanying images. A copy of the written consent is available for review by the Editor-in-Chief of this journal on request.

## Ethical approval

This research did not involve any human or animal subjects, or any other activity that might warrant ethical approval. Therefore, an ethical approval was not required for the completion of this paper.

## Funding

This study did not receive any specific grant from funding agencies in the public, commercial, or not-for-profit sectors.

## Guarantor

Fauriski Febrian Prapiska.

## CRediT authorship contribution statement

Fauriski Febrian Prapiska: Resources, Data Curation, Investigation, Supervision. Rizky An Nabil: Conceptualization, Methodology, Investigation. Syah Mirsya Warli, Bungaran Sihombing, Dhirajaya Dharma Kadar, and Ginanda Putra Siregar: Writing-Review & Editing, Supervision.

## Declaration of competing interest

All authors have no conflicts of interest to declare.
